# Overexpression of lncRNA SLC26A4‐AS1 inhibits papillary thyroid carcinoma progression through recruiting ETS1 to promote ITPR1‐mediated autophagy

**DOI:** 10.1111/jcmm.16545

**Published:** 2021-08-10

**Authors:** Dong Peng, Wenfa Li, Bojuan Zhang, Xuefen Liu

**Affiliations:** ^1^ Department of Nuclear Medicine Chongqing Rongchang People's Hospital Chongqing China; ^2^ Department of Cardiac Macrovascular Surgery Chongqing University Three Gorges Hospital/Chongqing Three Gorges Central Hospital Chongqing China; ^3^ Department of Oncology Chongqing University Three Gorges Hospital/Chongqing Three Gorges Central Hospital Chongqing China; ^4^ Department of Oncology Chongqing Rongchang People's Hospital Chongqing China

**Keywords:** autophagy, ITPR1, Long non‐coding RNA, papillary thyroid carcinoma, SLC26A4‐AS1, transcription factor

## Abstract

Papillary thyroid carcinoma (PTC), accounting for approximately 85% cases of thyroid cancer, is a common endocrine tumour with a relatively low mortality but an alarmingly high rate of recurrence or persistence. Long non‐coding RNAs (lncRNAs) is emerging as a critical player modulating diverse cellular mechanisms correlated with the progression of various cancers, including PTC. Herein, we aimed to investigate the role of lncRNA SLC26A4‐AS1 in regulating autophagy and tumour growth during PTC progression. Initially, ITPR1 was identified by bioinformatics analysis as a differentially expressed gene. Then, Western blot and RT‐qPCR were conducted to determine the expression of ITPR1 and SLC26A4‐AS1 in PTC tissues and cells, both of which were found to be poorly expressed in PTC tissues and cells. Then, we constructed ITPR1‐overexpressing cells and revealed that ITPR1 overexpression could trigger the autophagy of PTC cells. Further, we performed a series of gain‐ and loss‐of function experiments. The results suggested that silencing of SLC26A4‐AS1 led to declined ITPR1 level, up‐regulation of ETS1 promoted ITPR1 expression, and either ETS1 knockdown or autophagy inhibitor Bafilomycin A1 could mitigate the promoting effects of SLC26A4‐AS1 overexpression on PTC cell autophagy. In vivo experiments also revealed that SLC26A4‐AS1 overexpression suppressed PTC tumour growth. In conclusion, our study elucidated that SLC26A4‐AS1 overexpression promoted ITPR1 expression through recruiting ETS1 and thereby promotes autophagy, alleviating PTC progression. These finding provides insight into novel target therapy for the clinical treatment of PTC.

## 1 INTRODUCTION

Thyroid cancer is responsible for 567 000 annual cases worldwide as its incidence increases over years but its mortality is only less than 1%.^1^ Papillary thyroid carcinoma (PTC) accounts for approximately 85% of thyroid cancer cases, characterized by neck mass or thyroid enlargement with few copy‐number alterations, even usually without obvious symptoms.^2^ Since the etiology of the disease is not well understood, there are still debates over the PTC management including preoperative assessment and overtreatment.^3^ Application of molecular markers exerts promising effects on disease diagnosis and treatment,^4^ so it might guide the intensity of PTC treatment in 1 day and thus attract investigators' attentions.

Long non‐coding RNAs (lncRNAs), a kind of the non‐coding RNAs, have been highlighted as potential molecular biomarkers over the past 25 years.^5^, ^6^ Being highly heterogeneous transcripts, lncRNAs regulate gene expression by means of diverse mechanisms and affect various aspects of cellular homeostasis in cancers, including proliferation, autophagy or genomic stability.^7^ In PTC, regulatory role of lncRNAs, such as COMET and SNHG15, has also been implicated in cancer cell viability and other activities.^8^, ^9^ Recently, a newly discovered lncRNA, SLC26A4‐AS1, was suggested to potently inhibit RNA metabolic process, cell migration, and decline hormone levels in thyroid cancer.^10^ It is also demonstrated to exert antiangiogenic effects on human glioma by activating transcription factors.^11^ In line with previous studies, bioinformatics analysis of the current study also indicated up‐regulated expression of SLC26A4‐AS1 in PTC tissues. Nevertheless, the mechanism underlying SLC26A4‐AS1 in the progression of PTC remains elusive and it is meaningful to further explore its therapeutic potential for PTC management.

Autophagy is a lysosomal pathway for catabolism of intracellular material and is key to tissue homeostasis, adaptation to stress situations, immune responses.^12^ Due to these conserved functions, autophagy has been highlighted to act as an essential adaptation pathway that promotes tumourigenesis by regulating survival of cancer cells under stress, while tumour cell metabolism is controlled by autophagy.^13^ Inositol 1,4,5‐trisphosphate receptor, type 1 (ITPR1), as a direct novel target of HIF2α, has been indicated to induce autophagy and thereby exert protective effects on renal cancer cells against natural killer cells.^14^ ITPR1 has also been implicated as an autophagy‐related gene and a promising target for PTC,^15^ but the specific role in the progression in PTC remains uncharacterized. Herein, we aimed to investigate in the current study, whether SLC26A4‐AS1 interacts with ITPR1 and how SLC26A4‐AS1 overexpression alleviates the progression of PTC.

## 2 METHODS

### 2.1 Ethical statement

The experiment was approved by the Ethics Committee of Chongqing University Three Gorges Hospital and conducted in compliance with the *Declaration of Helsinki*. All individuals signed written informed consent documents. All animal experiments were performed with approval of the Animal Ethics Committee of Chongqing University Three Gorges Hospital. The experiments involving animals were conducted following the recommendations in the *Guide for the Care and Use of Laboratory Animals* of the National Institutes of Health.

### 2.2 Clinical specimens

Cancer tissues and corresponding adjacent normal tissues were collected from 30 PTC patients who underwent thyroidectomy in Chongqing University Three Gorges Hospital from January 2017 to December 2018. The tissues were stored in liquid nitrogen at −80°C. None of them had received chemotherapy, radiotherapy or immunotherapy. Cancer tissues of 20 PTC cases were subjected to immunohistochemical staining, fixed by formalin and paraffin‐embedded, with adjacent normal tissues as controls.

### 2.3 Cell culture

Human normal thyroid epithelial cells Nthy‐ori‐3‐1 and human PTC cell lines TPC‐1, BCPAP, K1 and IHH4 were purchased from American Type Culture Center (ATCC). Nthy‐ori‐3‐1 cells were cultured in F12K medium (Gibco) containing 20% FBS and the PTC cells were cultured in RPMI‐1640 (Gibco) containing 10% FBS at 37℃ with 5% CO_2_ and 95% air. A natural killer cell line (NK‐92) was cultured 300 U/mL recombinant human IL2 (rhIL2) RPMI medium.

Lentivirus was packaged using LV5‐GFP (lentivirus overexpression vector) and pSIH1‐H1‐copGFP (shRNA lentiviral vector) and SLC26A4‐AS1 shRNA, ETS1 shRNA, ITPR1 shRNA and shRNA of negative control (NC) designed by Shanghai GenePharma Co., Ltd. The packaged lentivirus and vectors were co‐transfected into HEK293T cells. After culture for 48 hours, the supernatant was collected and underwent centrifugation and filtration, where supernatant was detected for determining virus titre. Then the cells were transduced with lentiviral vectors carrying plasmids containing shRNA targeting SLC26A4‐AS1/ETS1 (sh‐SLC26A4‐AS1, sh‐ETS1), or SLC26A4‐AS1/ETS1 overexpression plasmids (oe‐SLC26A4‐AS1, oe‐ETS1), or corresponding controls. Cells in the logarithmic growth phase were collected, digested in trypsin and triturated into cell suspension (5 × 10^4^ cell/mL). The suspension was seeded onto 6‐well plates and incubated overnight. Then, 48 hours after transfection, RT‐qPCR was performed to determine gene expression in cells.

### 2.4 Immunohistochemistry (IHC)

Thyroid tissue sections (5 μm‐thick) were stained by using the ABC reagent kit (Vector Laboratories). ITPR1 expression in normal and malignant samples was semi‐quantitatively scored.^16^ The immunoreactivity was determined by the sum of the staining intensity and the area of positive staining according to negative (0), weak (1‐2), moderate (3), or strong (4‐6) staining.

### 2.5 Reverse transcription quantitative polymerase chain reaction (RT‐qPCR)

Total RNA from cells and tissues was extracted using Trizol Reagent (Invitrogen). RNA concentration and purity were determined using an ultraviolet spectrophotometer (ND‐1000, NanoDrop Technologies Inc). The RNA was reversely transcribed into cDNA with PrimeScript RT reagent Kit (Takara, Japan). Primer sequences were synthesized Guangzhou RiboBio Co., Ltd. (Guangdong, China), as shown in Table S1. The synthesized cDNA was subjected to RT‐qPCR based on the introductions of SYBR^®^ Premix Ex TaqTM II Kit (Tli RNaseH Plus). Real‐time PCR was performed using ABI PRISM^®^ 7900HT System (7500, ABI Company). With GAPDH as an internal reference, the relative expression level of target genes was calculated (2^−△△CT^ method).

### 2.6 Western blot analysis

PTC tissues and cells upon different treatments were washed in PBS, scraped in lysis buffer (C0481), and incubated at 4°C for 30 minutes. The lysate was collected to 1.5 mL EP tube and centrifuged at 12 000 g for 15 minutes. The supernatant was collected and protein concentration was determined by bicinchoninic acid (BCA) kit (Wuhan Boster). The protein was separated by 10% sodium dodecyl sulfate polyacrylamide gel electrophoresis and transferred onto polyvinylidene fluoride membrane (Millipore) which was blocked by 5% non‐fat dried milk for 1 hour. The membranes were incubated with primary antibodies overnight at 4°C and incubated with horseradish peroxidase secondary antibody (TransGen Biotech) for 1 hour. The membranes were developed using enhanced chemiluminescence (ECL; Baomanbio Co., Ltd.). The proteins were quantified with the grey value of each protein and internal reference GAPDH (1:1000, ab37168) for normalisation. Image J analysis software was applied to quantify band intensity. The primary antibodies from Abcam included: rabbit anti‐ITPR1 (1:1000, ab5804), rabbit anti‐ETS1 (1:1000, ab186844), rabbit anti‐LC3 (1:1000, ab48394), and rabbit anti‐p62 (1:1000, ab109012), rabbit anti‐Beclin‐1 (ab207612, 1:2000), and rabbit anti‐Bcl2 (ab196495, 1:250).

### 2.7 Immunofluorescence

Cells were seeded onto 24‐well plates (2 × 10^4^ cell/well), perfused with phosphate, and fixed with 4% paraformaldehyde. The cells were incubated with primary antibodies against LC3 (3868 CST) at a 1:200 dilution at 4°C overnight and with fluorescent secondary antibody (1:100 dilution; ZSGB‐BIO) for 2 hours. The cells were stained with diaminophenylindole (DAPI, Beyotime), and images were photographed under a confocal microscope (NIKON).

### 2.8 Transmission electron microscopy

TPC‐1 cells were centrifuged, fixed in 2.5% glutaraldehyde in 0.1 mol/L sodium cacodylate buffer, dehydrated in a gradient of ethanol, and embedded in epoxy resin. The embedded samples were sliced into sections (50‐60 nm) and the sections were stained with uranyl acetate and lead citrate and observed under a JEM‐1400 transmission electron microscope.

### 2.9 Dual‐luciferase reporter gene assay

ITPR1‐2Kb enhancer region was cloned to psiCHECK‐2 luciferase reporter gene plasmids. The plasmids were co‐transfected with oe‐negative control (NC), oe‐SLC26A4‐AS1, sh‐NC, and sh‐SLC26A4‐AS1 into TCL‐1 cells. 48 hours after co‐transfection, the cells were lysed with their Renilla luciferase activity and Firefly luciferase activity detected using luciferase detection kit (K801‐200, BioVision) and Dual‐Luciferase Reporter Gene System (Promega, Madison, WI, USA). The ratio of Firefly luciferase unit (FLU) divided by Renilla luciferase unit (RLU) values, reflected the activation of target gene.

### 2.10 RNA immunoprecipitation (RIP) assay

The RIP kit (Millipore Corporation) was adopted to examine the binding between SLC26A4‐AS1 and transcription factor ETS1. After washing in PBS, cells were lysed in lysis buffer for 10 minutes and centrifuged at 14 000 g for 10 minutes. Extracts were co‐precipitated with antibodies ETS1 (Cell Signaling Technologies, #14069) or IgG (ab172730, 1:100, Abcam). Briefly, 50 μL of magnetic beads were reset by adding 100 μL RIP Wash Buffer and incubated with antibodies for 30 minutes. Bead‐antibody complex was suspended in 900 μL of RIP Wash Buffer and incubated with 100 μL of cell extract at 4°C overnight followed by collection of bead‐antibody complex. The RNA was purified by proteinase K buffer for RT‐qPCR analysis.

### 2.11 Chromatin immunoprecipitation (ChIP)

When confluence reached 70‐80%, cells were fixed with 1% formaldehyde at room temperature for 10 minutes for crosslinking cell DNA and protein. Then the cells sonicated for 10 seconds, 15 cycles. After centrifugation at 13 000 rpm, the supernatant was divided into two parts and respectively incubated with anti‐rabbit IgG (ab109489, 1:100, Abcam) and target protein specific anti‐mouse ETS1 (Cell Signaling: #14069). Protein Agarose/Sepharose was added to precipitate endogenous DNA‐protein complex, followed by centrifugation and decrosslinking. DNA fragments were purified with Phenol/Chloroform. ITPR1 enhancer specific primer: Forward: GCGTCCAGTGACCAGGG, Reverse: TTAAAGCGGCTCCGGGTG was used to evaluate the enrichment of ETS1 in ITPR1 enhancer. Same procedure was performed to detect enrichment of SLC26A4‐AS1 in ETS1 enhancer.

### 2.12 Tumor xenograft model

A total of 40 BALB/c mice (18‐21 g, 5 week‐old, male) were maintained in a specific pathogen free condition. The TPC‐1 cells (4 × 10^6^) transfected with oe‐NC, pcDNA3.1 plasmid vector, oe‐ITPR1, oe‐SLC26A4‐AS1, and oe‐SLC26A4‐AS1 + sh‐ETS1 were subcutaneously injected into mice (n = 8). Four days after inoculation, we began to measure volume of tumour. Then, mice were euthanized with excess CO_2_. The volume (V) of xenograft tumour was calculated using the formula: V = (A × B^2^)/2 (A, long diameter; B, short diameter). The mean volume at each hour was presented in the curve.

### 2.13 Statistical analysis

The data were processed using SPSS 21.0 statistical software (IBM SPSS Statistics). Measurement data were expressed as mean ± standard deviation. The cancer tissues and adjacent normal tissues were compared by paired *t* test while analysis of the other two group was performed through unpaired *t* test. Analysis among multiple groups was conducted by one‐way analysis of variance (ANOVA) followed by Tukey's post hoc test. Data at different time points among groups were compared by repeated measures ANOVA, followed by Bonferroni. *P* < .05 was considered statistically significant. All experiments were repeated at least three times.

## 3 RESULTS

### 3.1 ITPR1 is down‐regulated in PTC tissues and cells

**FIGURE 1:**
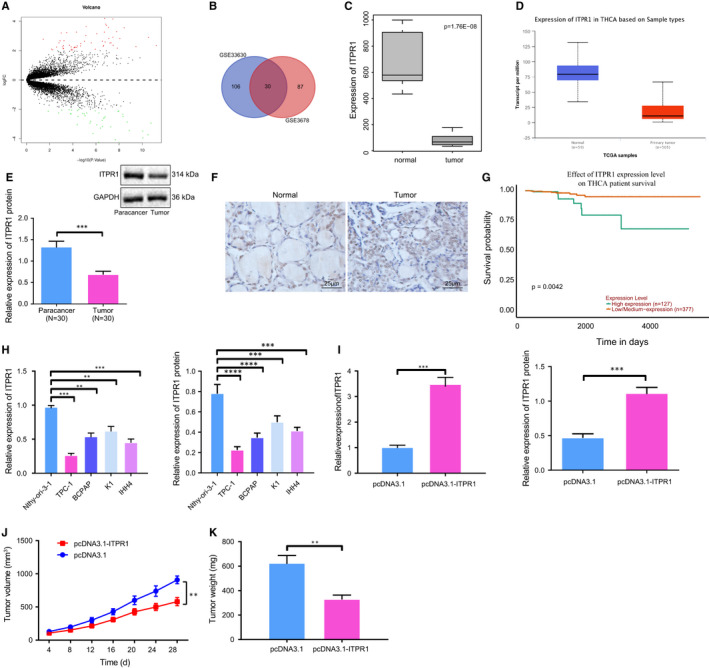
ITPR1 is poorly expressed in PTC tissues and cells and alleviates tumour growth. A, Volcano plot of differentially expressed genes in PTC. Abscissa: logFC; Ordinate: log10 p value. A red dot refers to up‐regulated gene; a green dot refers to down‐regulated genes. B, Venn diagram of PTC differentially expressed genes from profiling microarrays GSE33630 and GSE3678. Number refers to amount of genes. C, Box plot of ITPR1 expression in PTC samples and normal samples from GSE3678. D, Expression level of ITPR1 in PTC predicted by Ualcan database. E, RT‐qPCR and Western blot analyses of ITPR1 expression in 30 paired PTC tissues and adjacent normal tissues. *** *P* < .001 *vs*. adjacent normal tissues F, IHC of ITPR1 expression in PTC tissues and adjacent normal tissues (×400). G, RFS survival analysis of ITPR1 and prognosis of PTC patients. H, RT‐qPCR and Western blot analyses of ITPR1 expression in TPC‐1, BCPAP, K1, IHH4, and Nthy‐ori‐3‐1 cells. **P < .01, and *** *P* < .001 *vs*. Nthy‐ori‐3‐1 cells. I, RT‐qPCR and Western blot analyses of ITPR1 expression level in TPC‐1 cells upon transfection of oe‐ITPR1. **P < .01 versus pcDNA3.1. J, Quantification of mouse tumour after injection of oe‐ITPR1 cells and quantification of tumour volume within days after treatment. K, Quantification of tumour weight after injection of oe‐ITPR1 cells. * *P* < .05, **P < .01, and *** *P* < .001 versus pcDNA3.1. Measurement data were expressed as mean ± standard deviation. The cancer tissues and adjacent normal tissues were compared by paired *t* test while analysis of the other two group was performed through unpaired *t* test. Analysis among multiple groups was conducted by ANOVA followed by Tukey's post hoc test. Data at different time points among groups were compared by repeated measures ANOVA, followed by Bonferroni

For exploring the molecular mechanism of PTC, we screened differentially expressed genes on microarray (profiling microarray GSE3678) from Gene Expression Omnibus (GEO) and obtained 117 differentially expressed genes including 57 up‐regulated genes and 60 down‐regulated genes (Figure 1A). Another 136 differentially expressed genes were obtained from microarray GSE33630, consisting of 70 up‐regulated genes and 66 down‐regulated genes, while 30 genes were gained from intersection of microarrays GSE33630 and GSE3678 (Figure 1B). Differentially expressed gene expression analysis of the 30 genes indicated ITPR1 was down‐regulated in PTC (Figure 1C), in line with the prediction result from Ualcan database (Figure 1D). A previous study has noted ITPR1 as a candidate gene of B‐RAF kinase.^17^ Nevertheless, the underlying mechanism of ITPR1 in PTC remains uncharacterized. In the present study, RT‐qPCR analysis was used to determine ITPR1 in 30 paired PTC tissues and adjacent normal tissues, while five paired tissues were subjected to Western blot analysis. As a result, ITPR1 protein and mRNA expression of ITPR1 was down‐regulated in PTC tissues (Figure 1E). IHC confirmed the lower expression in PTC tissues (Figure 1F). According to relapse‐free survival (RFS) analysis, low ITPR1 expression was positively associated with poor prognosis of PTC patients (Figure 1G). In vitro experiments further validated these findings. ITPR1 was poorly expressed in PTC cells TPC‐1, BCPAP, K1, and IHH4, especially in TPC‐1 cells, as compared with normal thyroid epithelial cell Nthy‐ori‐3‐1 (Figure 1H). ITPR1 overexpression plasmids were then transfected into TPC‐1 cells. As Western blot analysis confirmed the efficiency of transfection (Figure 1I), the oe‐ITPR1‐treated cells were subcutaneously injected into nude mice and the weight and size of xenografted tumours were measured. As a consequence, tumour growth was suppressed as reflected by decreased weight and volume (Figure 1J,K). Taken together, ITPR1 was down‐regulated in PTC and its overexpression suppressed PTC tumourigenesis.

### 3.2 Overexpression of ITPR1 promotes autophagy of PTC cells

**FIGURE 2:**
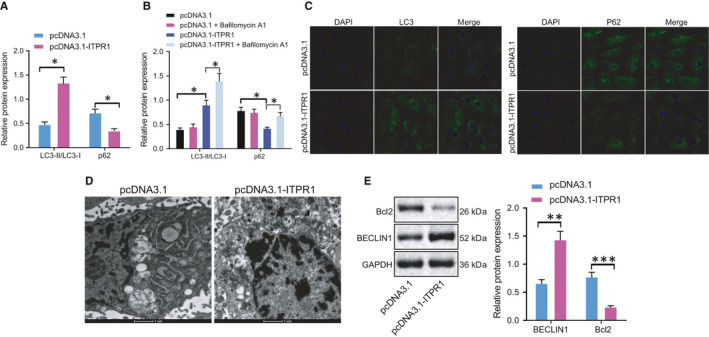
Overexpression of ITPR1 contributes to autophagy in PTC. A, Western blot analysis of autophagy related protein LC3‐I, LC3‐II, and p62 expression in oe‐ITPR1‐treated cells. B, Western blot analysis of autophagy related protein LC3‐I, LC3‐II, and p62 expression in oe‐ITPR1‐treated cells upon Bafilomycin A1 treatment. C, Immunofluorescence of LC3 and p62 expression level in oe‐ITPR1‐treated cells (×400). D, Representative images of transmission electron microscope of autophagosome in oe‐ITPR1‐treated cells. E, Western blot analysis of Beclin‐1 and Bcl2 expression upon overexpression of ITPR1. * *P* < .05, **P < .01, and *** *P* < .001. Measurement data were expressed as mean ± standard deviation. The cancer tissues and adjacent normal tissues were compared by paired *t* test while analysis of the other two group was performed through unpaired *t* test. Analysis among multiple groups was conducted by ANOVA followed by Tukey's post hoc test

Previously, ITPR1 has been reported to promote autophagy and thus sensitize paclitaxel cytotoxicity in human cancers.^18^ Herein, we aimed to determine whether ITPR1 was involved in cell autophagy in PTC cells and detected expression of autophagy‐related proteins (LC3‐I, LC3‐II, and p62) by Western blot analysis. In response to ITPR1 overexpression in TPC‐1 cells, p62 expression reduced, with the value of LC3‐II/LC3‐I elevated (Figure 2A). Then, autophagy inhibitor Bafilomycin A1 was applied to treat the cells expressing empty pcDNA3.1 plasmid vector or ITPR1 overexpression cells, followed by determination of LC3‐1, LC3‐II, and p62 expression. Results showed that Bafilomycin A1 alone hardly altered the ratio of LC3‐II/LC3‐I and p62 expression; and its combination with ITPR1 overexpression further elevated the ratio of LC3‐II/LC3‐I that was already up‐regulated by ITPR1 overexpression alone, but reversed the down‐regulation of p62 level induced by ITPR1 overexpression alone (Figure 2B). Immunofluorescence depicted that overexpression of ITPR1 enhanced LC3‐II and decreased p62 expression, promoting autophagy formation (Figure 2C), consistent with the results from transmission electron microscope where the amount of autophagosome increased (Figure 2D). Additionally, another two autophagy marker genes, Beclin‐1 and Bcl2, were detected in the treated cells. Results from Western blot analysis unravelled that ITPR1 overexpression led to elevated Beclin‐1 protein level and down‐regulated Bcl2 level (Figure 2E). Collectively, the evidence indicated that up‐regulation of ITPR1 could significantly facilitate PTC cell autophagy.

### 3.3 SLC26A4‐AS1 promotes ITPR1 expression by recruiting transcription factor ETS1

**FIGURE 3:**
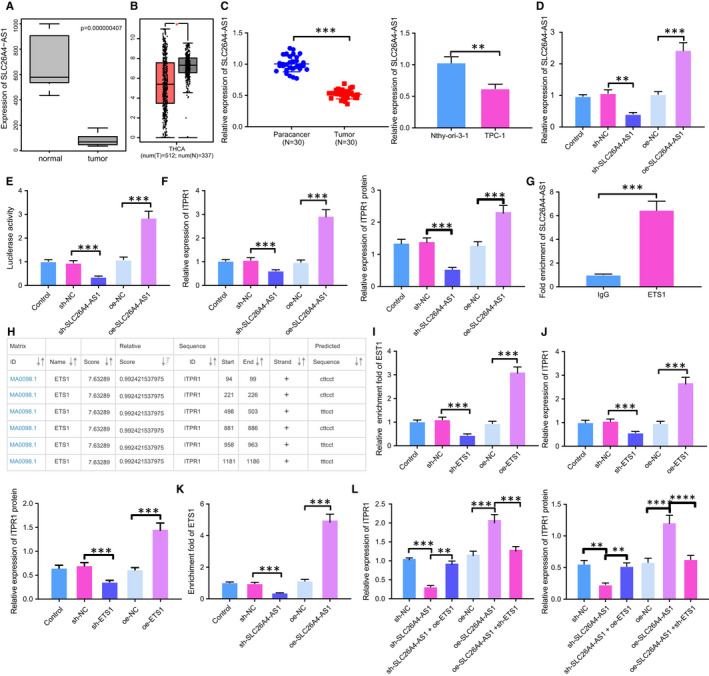
SLC26A4‐AS1 up‐regulates ETS1 expression and thereby promotes ITPR1 expression. A, Box plot of ITPR1 expression in PTC samples and normal samples from GSE3678. B, Box plot of ITPR1 expression in PTC samples and normal samples from TCGA database. Red dots refer to tumour sample, green dots refer to normal samples. C, RT‐qPCR analysis of SLC26A4‐AS1 expression in PTC tissues and TPC‐1 cells. D, RT‐qPCR analysis of SLC26A4‐AS1 expression in TPC‐1 cells upon treatment of oe‐SLC26A4‐AS1 or sh‐SLC26A4‐AS1. E, Dual‐luciferase reporter gene assay of ITPR1 promoter activity upon treatment of oe‐SLC26A4‐AS1 or sh‐SLC26A4‐AS1. F, Western blot and RT‐qPCR analyses of ITPR1 mRNA and protein expression upon treatment of oe‐SLC26A4‐AS1 or sh‐SLC26A4‐AS1. G, RIP of fold enrichment of SLC26A4‐AS1 upon treatment with ETS1 or IgG. H, Binding site between ETS1 and ITPR1 predicted by JASPER database. I, ChIP of fold enrichment of ITPR1 enhancer upon treatment of oe‐ETS1 or sh‐ETS1. J, RT‐qPCR and Western blot analyses of ITPR1 mRNA and protein expression upon treatment of oe‐ETS1 or sh‐ETS1. K, ChIP of fold enrichment of ITPR1 promoter upon treatment of oe‐ETS1 or sh‐ETS1. L, RT‐qPCR and Western blot analyses of ITPR1 mRNA and protein expression in TPC‐1 cells after treatment of sh‐SLC26A4‐AS1 + oe‐ETS1, oe‐SLC26A4‐AS1 + sh‐ETS1, or controls. * *P* < .05, **P < .01, and *** *P* < .001. Measurement data were expressed as mean ± standard deviation. The cancer tissues and adjacent normal tissues were compared by paired *t* test while analysis of the other two group was performed through unpaired *t* test. Analysis among multiple groups was conducted by ANOVA followed by Tukey's post hoc test

According to lncMAP database, SLC26A4‐AS1, ITPR1 and ETS1 were indicated to form triplet (Table S2). SLC26A4‐AS1 has been implicated in the positive prognosis of breast cancer and PTC patients, as it is down‐regulated in cancer cells.^10^, ^19^ In line with previous studies, our work indicated that SLC26A4‐AS1 was down‐regulated in PTC in microarray GSE3678 (Figure 3A). Prediction of GEPIA also showed low SLC26A4‐AS1 expression in PTC (Figure 3B). To confirm the prediction, we then detected SLC26A4‐AS1 expression in cells and tissues. Results from RT‐qPCR analysis confirmed the low SLC26A4‐AS1 expression in PTC cells and tissues (Figure 3C). Upon construction of plasmids overexpressing or silencing SLC26A4‐AS1, RT‐qPCR analysis determined the efficiency of transfection (Figure 3D). Moreover, in the presence of SLC26A4‐AS1 overexpression, ITPR1 promoter was activated and ITPR1 expression was augmented, while SLC26A4‐AS1 interference led to the opposite (Figure 3E,F). Results of RIP demonstrated that compared to IgG, ETS1 could bind to greater SLC26A4‐AS1, suggesting the binding between SLC26A4‐AS1 and ETS1 (Figure 3G). Binding site between ETS1 and ITPR1 was indicated by JASPER database (Figure 3H). To confirm the binding site, we detected the enrichment fold of ETS1 in the promoter region of ITPR1 by ChIP experiment. In the presence of oe‐ETS1, higher enrichment fold of ETS1 was observed in the promoter region, indicating the greater binding between ETS1 and ITPR1, while silencing of ETS1 inhibited their binding (Figure 3I). We found that silencing of ETS1 led to ITPR1 down‐regulation while up‐regulation of ETS1 induced ETS1 expression in PTC cells (Figure 3J). To further elucidate the interaction between SLC26A4‐AS1, ITPR1 and ETS1, we treated TPC‐1 with plasmids expressing SLC26A4‐AS1 and performed ChIP to detect the enrichment level of ETS1. Upon treatment with silencing of SLC26A4‐AS1, the enrichment of ETS1 declined and the advent of oe‐SLC26A4‐AS1 increased the enrichment (Figure 3K), indicating that SLC26A4‐AS1 might recruit ETS1 to regulate ITPR1 expression. Further, results from Western blot and RT‐qPCR analyses revealed that overexpression of ETS1 in sh‐SLC26A4‐AS1‐treated cells resulted in elevated ITPR1 expression and that silencing of ETS1 declined ITPR1 expression (Figure 3L). Taken altogether, these data suggested that SLC26A4‐AS1 recruited ETS1 to promote ITPR1 expression in PTC cells.

### 3.4 SLC26A4‐AS1 promotes ITPR1 through recruiting ETS1 and thereby enhances autophagy

**FIGURE 4:**
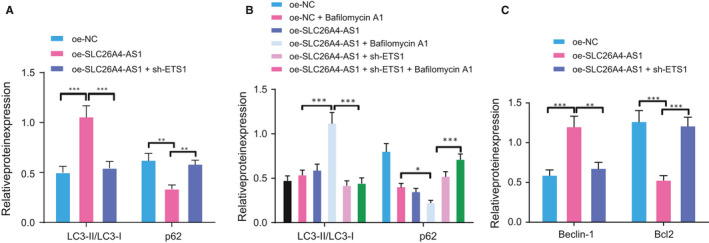
Overexpression of SLC26A4‐AS1 induces autophagy in PTC through promoting ETS1. A, Western blot analysis of autophagy related protein LC3‐I, LC3‐II, and p62 expression in oe‐ITPR1‐treated cells. B, Western blot analysis of autophagy related protein LC3‐I, LC3‐II, and p62 expression in oe‐SLC26A4‐AS1‐treated cells upon Bafilomycin A1. C, Western blot analysis of Beclin‐1 and Bcl2 expression upon oe‐SLC26A4‐AS1. * *P* < .05, **P < .01, and *** *P* < .001. Measurement data were expressed as mean ± standard deviation. The cancer tissues and adjacent normal tissues were compared by paired *t* test while analysis of the other two group was performed through unpaired *t* test. Analysis among multiple groups was conducted by ANOVA followed by Tukey's post hoc test

To detect the effort verified interaction between SLC26A4‐AS1, ITPR1 and ETS1 on autophagy in PTC, we detected autophagy‐related protein expression in the presence of SLC26A4‐AS1 overexpression and ETS1 knockdown by Western blot analysis. The results indicated that overexpression of SLC26A4‐AS1 increased LC3‐II expression and reduced p62 expression with LC3‐II/LC3‐I increased, but additional treatment with sh‐ETS1 decreased LC3‐II expression and increased p62 expression, while LC3‐I expression was hardly altered (Figure 4A). The presence of autophagy inhibitor Bafilomycin A1, suppressed the autophagy induced by oe‐SLC26A4‐AS1, not affecting LC3‐II level but increasing p62 expression, and Bafilomycin A1 also increased p62 level in cells overexpressing SLC26A4‐AS1 and silencing ETS1 (Figure 4B). Additionally, Western blot measurement of the expression of another two autophagy marker gene, Beclin‐1 and Bcl2 uncovered increased Beclin‐1 and decreased Bcl2 in response to SLC26A4‐AS1 overexpression (Figure 4C). Altogether, these results indicated that up‐regulation of SLC26A4‐AS1 could significantly facilitate PTC cell autophagy.

### 3.5 SLC26A4‐AS1‐ETS1‐ITPR1 axis is associated with PTC tumour growth

**FIGURE 5:**
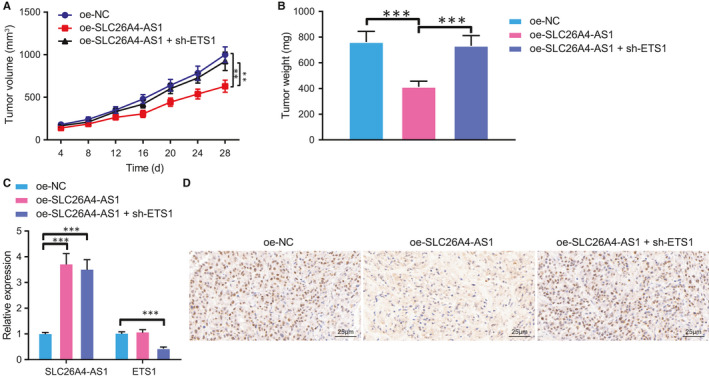
SLC26A4‐AS1 represses tumour growth through up‐regulation of ITPR1 by ETS1. A, Representative macroscopic images of mouse tumour upon treatment of oe‐SLC26A4‐AS1, oe‐SLC26A4‐AS1 + sh‐ETS1 or controls. B, Quantification of tumour weight upon treatment of oe‐SLC26A4‐AS1, oe‐SLC26A4‐AS1 + sh‐ETS1 or controls. C, RT‐qPCR analysis of SLC26A4‐AS1 and ETS1 expression in oe‐SLC26A4‐AS1‐ and sh‐ETS1‐treated mice and or controls. D, Immunohistochemistry of ITPR1 protein expression upon all treatments (×400). * *P* < .05 *vs*. oe‐NC, ** *P* < .01, and *** *P* < .001. Measurement data were expressed as mean ± standard deviation. Analysis among multiple groups was conducted by ANOVA followed by Tukey's post hoc test. Data at different time points among groups were compared by repeated measures ANOVA, followed by Bonferroni

To further investigate whether SLC26A4‐AS1‐ETS1‐ITPR1 axis controls tumour growth in vivo, we established mouse model with altered expression of SLC26A4‐AS1 and ETS1. Our data revealed that SLC26A4‐AS1 overexpression reduced tumour volume and weight while its combination with ETS1 knockdown abrogated the reduction (Figure 5A,B). RT‐qPCR demonstrated that either SLC26A4‐AS1 overexpression alone or its combination with ETS1 interference effectively increased SLC26A4‐AS1 expression, while the latter also down‐regulated ETS1 expression (Figure 5C). Interestingly, IHC results then indicated elevated ITPR1 expression in SLC26A4‐AS1 overexpression mice, whereas silencing of ETS1 reversed the alteration (Figure 5D). Taken together, our data elucidated that SLC26A4‐AS1 promoted autophagy and suppressed PTC tumour growth through ETS1‐mediated ITPR1 regulation.

## 4 DISCUSSION

Most of PTC incident cases are papillary in origin and are both small and localized, whilst survival rate of patients with these small localized PTC achieves as high as 99% at 20 years.^20^ Incidence of PTC has increased rapidly in the past 15 years probably due to increasing detection, as the reasons are not completely understood.^21^ Although the great improvement has successfully declined the risk of the condition, further investigations are still required to achieve more precise treatment and avoid overtreatment. The present study centred on the potential value of SLC26A4‐AS1 in PTC treatment and demonstrated that SLC26A4‐AS1 overexpression protected against PTC malignancy by recruiting ETS1 to enhance ITPR1 expression.

LncRNAs generally modulate autophagy *via* regulating the expression of related genes, functioning as competing endogenous RNAs (ceRNAs) to modulate autophagyrelated microRNAs (miRNAs), where autophagy also may in turn affect lncRNA expression.^22^ The interaction between lncRNAs and autophagy depends on specific cellular environmental stress and tumour microenvironment.^23^ LncRNA GAS8‐AS1 inhibited proliferation and activated autophagy leading to cell death with increased autophagosomes in PTC, by promoting expression of autophagy‐related gene 5.^24^ Consistently, our work indicated that SLC26A4‐AS1 suppressed PTC progression by enhancing autophagy‐related protein expression. The role of SLC26A4‐AS1 actually has been stressed before. SLC26A4‐AS1 is implicated associated with PTC patient disease free survival.^25^ Moreover, the study by Wang et al noticed that the overexpression of lncRNA SLC26A4‐AS1 exerted anti‐oncogenic effect on PTC, reducing tumour volume and promoting apoptosis.^26^ Unlike Wang's findings, autophagy and related genes ITPR1 as well as ETS1 were highlighted in the current study, whilst SLC26A4‐AS1 enhanced autophagy by recruiting ETS1 and thereby exerted protective effect on PTC cells.

Apart from lncRNAs, transcription factors are also a hot spot of drug targets for their roles in cellular processes.^27^ Mutated or dysregulated transcription factors serve as drivers of cancer where they mediate aberrant gene expression, blockade of differentiation and autophagy through various direct mechanisms.^28^ The thyroid transcription factors are fundamental to proper formation and normal function of the thyroid whilst transcription factors variations tend to trigger.^29^ ETS1, as a transcription factor, is frequently reported to promote cancer progression, such as hepatocellular carcinoma.^30^ In lung cancer, ETS1 contributes to transactivation of Twist1, a gene involved in tumour cell motility and dissemination, thereby worsening the condition.^31^ It is also indicated as a member of thyroid tumour‐oncogenic driver dysregulation in PTC,^32^ but the role is not clarified clearly. The interaction between ETS1 and lncRNA was previously noted in lung cancer.^33^ In the current study, silencing of ETS1 exhibited similar effect as Bafilomycin A1, suppressing autophagy‐induced by overexpression of SLC26A4‐AS1.

Autophagy influences cell survival through maintenance of cell bioenergetics and clearance of protein aggregates and damaged organelles.^34^ As for the effect of autophagy, it is context‐dependent and the clinical response to autophagy alteration varies widely.^35^ Autophagy can stimulate tumour antigen cross‐presentation while autophagy inhibition impinges on a normal immune response.^36^ Autophagy protects cells against internal and external stress, shaping cellular immunity.^37^ The activation of autophagy induced by ITPR1 in renal cell carcinoma results in the degradation of serine protease and impairs tumour cell killing, alleviating the disease progression.^38^ ITPR1‐induced autophagy was also reported in other tumours, including PTC where ITPR1 was identified as a differential expressed gene correlated with prognosis.^15^, ^17^ The clinical syndromes could be associated with ITPR1‐IgG and autoimmunity to ITPR1 may underlie diseases.^39^ In the current study, in the presence of overexpression of ITPR1, autophagy was enhanced, accompanied by decreased tumour volume and greater prognosis. Since the reaction was complex, involving other gene expression, further experiments are required to identify the specific role of autophagy in PTC progression and investigate the direct effects of the down‐regulated expression of SLC26A4‐AS1 on PTC development.

## 5 CONCLUSION

**FIGURE 6:**
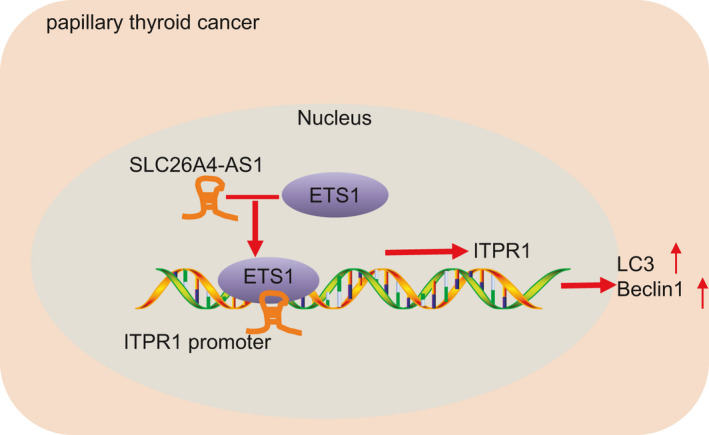
The underlying mechanism of SLC26A4‐AS1 in PTC. SLC26A4‐AS1 protected against malignant phenotypes of PTC cells by enhancing ITPR1 expression to recruit ETS1

In conclusion, our studies demonstrated that overexpression SLC26A4‐AS1 could promote autophagy and alleviate PTC development through promoting ITPR1 expression by recruiting ETS1 (Figure 6). These findings might underlie novel treatment against PTC in the future.

## CONFLICTS OF INTEREST

The authors declare no conflict of interest.

## AUTHOR CONTRIBUTIONS

**Dong Peng:** Conceptualization (equal); Investigation (equal); Methodology (equal); Resources (equal); Software (equal); Writing‐original draft (lead); Writing‐review & editing (equal). **Wenfa Li:** Data curation (lead); Formal analysis (lead); Project administration (lead); Validation (lead); Visualization (lead); Writing‐review & editing (equal). **Bojuan Zhang:** Data curation (supporting); Formal analysis (supporting); Resources (equal); Software (equal); Writing‐original draft (supporting); Writing‐review & editing (equal). **Xuefen Liu:** Data curation (supporting); Formal analysis (supporting); Resources (equal); Software (equal); Writing‐original draft (supporting); Writing‐review & editing (equal).

## Supporting information

Table S1‐S2

## Data Availability

Research data are not shared.
