# Overexpression of lncRNA SLC26A4‐AS1 inhibits papillary thyroid carcinoma progression through recruiting ETS1 to promote ITPR1‐mediated autophagy

**DOI:** 10.1111/jcmm.17293

**Published:** 2022-05-07

In Xuefen Liu et al.^1^ the immunofluorescence diagram pcDNA30.1 in Figure 2C, the WB experiments in Figure 1E,F are incorrect. The correct figure is shown below. The authors confirm all results, conclusions of this article remain unchanged.

**FIGURE 1:**
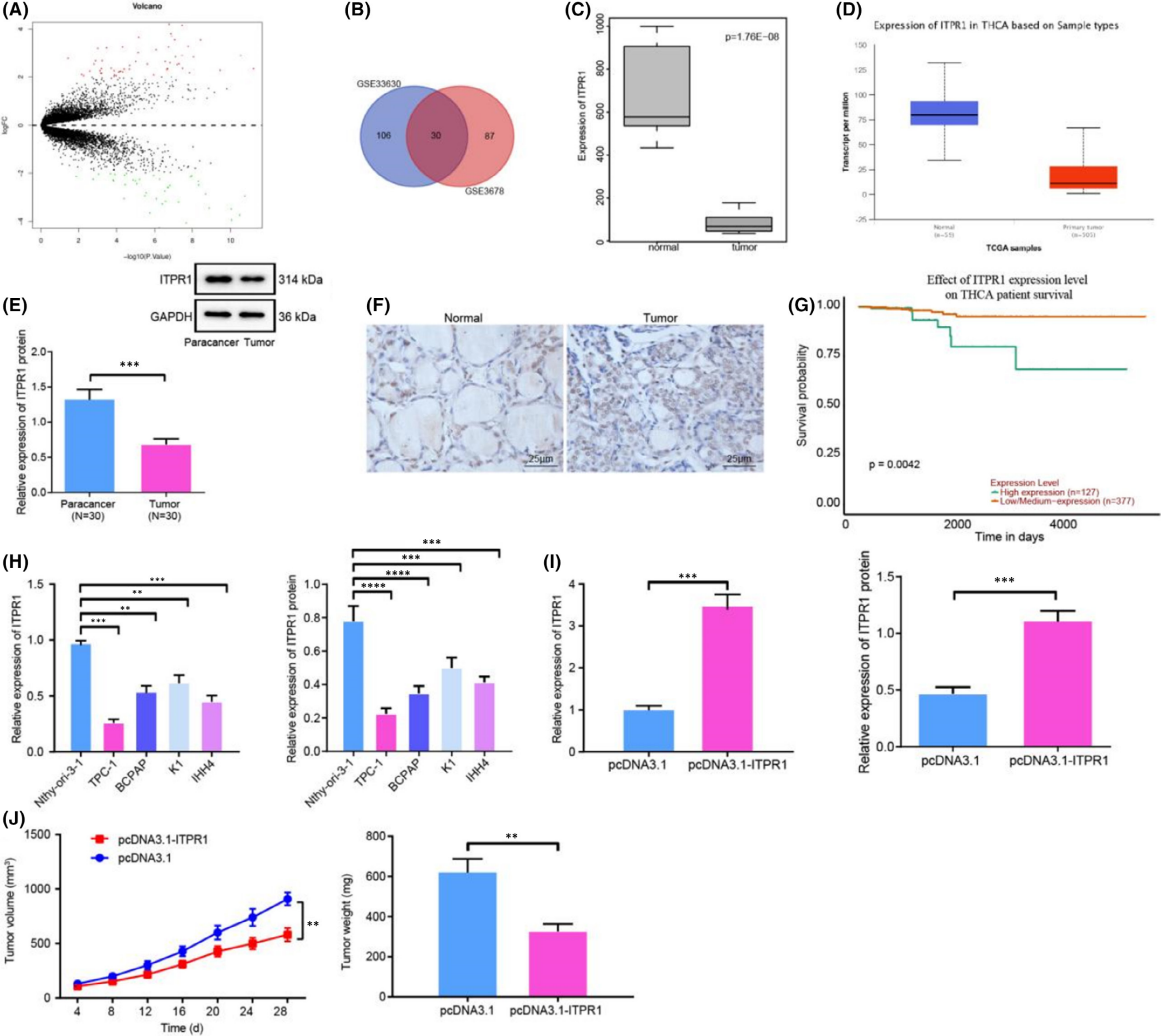
ITPR1 is poorly expressed in PTC tissues and cells and alleviates tumour growth. (A) Volcano plot of differentially expressed genes in PTC. Abscissa: logFC; Ordinate: log10 *p* value. A red dot refers to up‐regulated gene; a green dot refers to down‐regulated genes. (B) Venn diagram of PTC differentially expressed genes from profiling microarrays GSE33630 and GSE3678. Number refers to amount of genes. (C) Box plot of ITPR1 expression in PTC samples and normal samples from GSE3678. (D) Expression level of ITPR1 in PTC predicted by Ualcan database. (E) RT‐qPCR and Western blot analyses of ITPR1 expression in 30 paired PTC tissues and adjacent normal tissues. ***p < 0.001 versus adjacent normal tissues (F) IHC of ITPR1 expression in PTC tissues and adjacent normal tissues (400×). (G) RFS survival analysis of ITPR1 and prognosis of PTC patients. (H) RT‐qPCR and Western blot analyses of ITPR1 expression in TPC‐1, BCPAP, K1, IHH4 and Nthy‐ori‐3‐1 cells. **p < 0.01, and ***p < 0.001 versus Nthy‐ori‐3‐1 cells. (I) RT‐qPCR and Western blot analyses of ITPR1 expression level in TPC‐1 cells upon transfection of oe‐ITPR1. **p < 0.01 versus pcDNA3.1. (J) Quantification of mouse tumour after injection of oe‐ITPR1 cells and quantification of tumour volume within days after treatment. (K) Quantification of tumour weight after injection of oe‐ITPR1 cells. *p < 0.05, **p < 0.01, and ***p < 0.001 versus pcDNA3.1. Measurement data were expressed as mean ± standard deviation. The cancer tissues and adjacent normal tissues were compared by paired *t*‐test while analysis of the other two group was performed through unpaired *t*‐test. Analysis among multiple groups was conducted by ANOVA followed by Tukey's post hoc test. Data at different time points among groups were compared by repeated measures ANOVA, followed by Bonferroni

**FIGURE 2:**
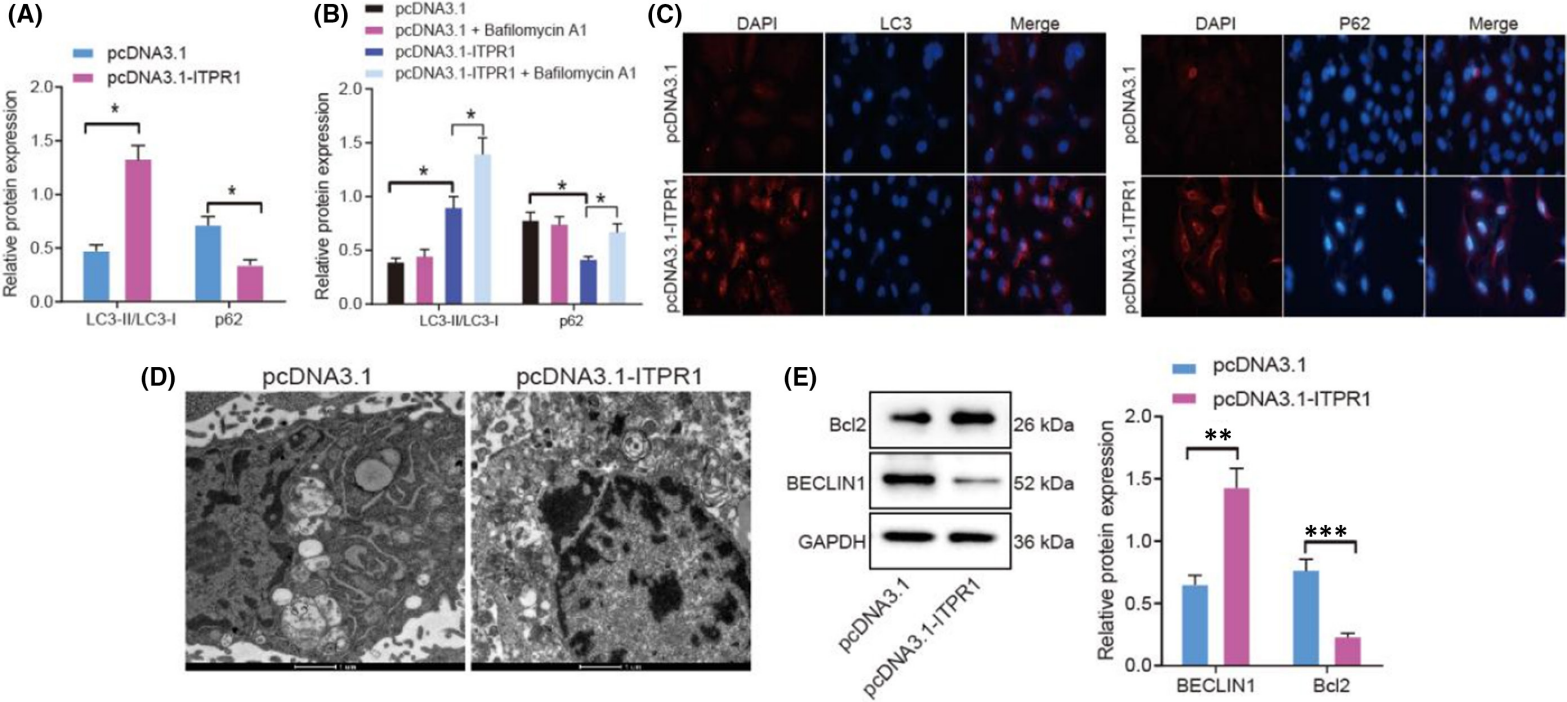
Overexpression of ITPR1 contributes to autophagy in PTC. (A) Western blot analysis of autophagy‐related protein LC3‐I, LC3‐II and p62 expression in oe‐ITPR1‐treated cells. (B) Western blot analysis of autophagy‐related protein LC3‐I, LC3‐II and p62 expression in oe‐ITPR1‐treated cells upon Bafilomycin A1 treatment. (C) Immunofluorescence of LC3 and p62 expression level in oe‐ITPR1‐treated cells (400×). (D) Representative images of transmission electron microscope of autophagosome in oe‐ITPR1‐treated cells. (E) Western blot analysis of Beclin‐1 and Bcl2 expression upon overexpression of ITPR1. *p < 0.05, **p < 0.01 and ***p < 0.001. Measurement data were expressed as mean ± standard deviation. The cancer tissues and adjacent normal tissues were compared by paired *t*‐test while analysis of the other two group was performed through unpaired *t*‐test. Analysis among multiple groups was conducted by ANOVA followed by Tukey's post hoc test
