# Supplementary material for: Overexpression of lncRNA SLC26A4‐AS1 inhibits papillary thyroid carcinoma progression through recruiting ETS1 to promote ITPR1‐mediated autophagy
Source: J Cell Mol Med. 2021 Aug 10;25(17):8148–58. doi: 10.1111/jcmm.16545 (PMC8419164; doi:10.1111/jcmm.16545)
Supplement: Supplementary file 1 — Table S1‐S2 [file JCMM-25-8148-s001.docx]

**Supplementary Table 1** Primer sequence for RT-qPCR

| Gene | Sequence |
| --- | --- |
| ITPR1 | Forward: 5´-ACCCAGGAACTGATATGTAAA-3´ |
|  | Reverse: 5´-TTTACATATCAGTTCCTGGGT-3´ |
| ETS1 | Forward: 5´-TCACTAAAGAACAGCAACGA-3´ |
|  | Reverse: 5´-GGTTTCACATCCTCTTTCTG-3´ |
| SLC26A4-AS1 | Forward: 5´-CTTGGCAATGGTTTGCTGGT-3´ |
|  | Reverse: 5´-AGATGGGCTCTGCCTGAGAAA-3´ |
| GAPDH | Forward: 5´-ACAACTTTGGTATCGTGGAAGG-3´ |
|  | Reverse: 5´-GCCATCACGCCACAGTTTC-3´ |

Note: ITPR1, Inositol 1,4,5-trisphosphate receptor, type 1; ETS1, transcription factor ETS proto-oncogene 1; GAPDH, Glyceraldehyde-3-phosphate dehydrogenase;

**Supplementary Table 2** LncRNA-TF-Gene triplet predicted by lncMAP database

| LncRNA Symbol | TF Symbol | Gene Symbol | Mediated pattern |
| --- | --- | --- | --- |
| SLC26A4-AS1 | ETS1 | ITPR1 | + - |
